# A randomized double-blind placebo-controlled multicenter trial of Bushen Yisui and Ziyin Jiangzhuo formula for constipation in Parkinson disease

**DOI:** 10.1097/MD.0000000000021145

**Published:** 2020-07-10

**Authors:** Zhaohui Jin, Zhengtang Liu, Lei Kang, Aoran Yang, Hongbo Zhao, XiaoYan Yan, Tianqing Zhang, Lei Gao, Aixian Liu, Boyan Fang

**Affiliations:** a Parkinson Medical Center, Beijing Rehabilitation Hospital, Capital Medical University; b Geriatrics Department, China Academy of Chinese Medical Sciences Xiyuan Hospital; c Medical Insurance Office, Dongzhimen Hospital of Beijing University of Chinese Medicine; d Traditional Chinese Medicine Rehabilitation Center, Beijing Rehabilitation Hospital, Capital Medical University; e Peking University Clinical Research Institute, Peking University First Hospital; f Neurology Department, Beijing Longfu Hospital, Beijing, China.

**Keywords:** complementary therapy, constipation, Parkinson disease, traditional Chinese medicine

## Abstract

**Introduction:**

Constipation is a common nonmotor symptom of Parkinson disease (PD). Constipation can also impact patient's quality of life. Chinese herbal medicines have been used for the treatment of constipation in PD. This trial will evaluate the efficacy and safety of a Chinese herbal formula Bushen Yisui and Ziyin Jiangzhuo (BYZJ) for the treatment of constipation in PD.

**Methods and analysis:**

This randomized, double-blind, placebo-controlled, multicenter clinical trial will involve 4 hospitals in Beijing, China. The study will aim to recruit 90 PD patients with constipation between 30 and 80 years-of age with a score of 1 – 4 on the Hoehn and Yahr scale. Once recruited, Patients will be randomized into a BYZJ group or a placebo group in a 2:1 ratio. The trial will include a 1-week run-in period, a 4-week double-blind treatment period, a 4-week and a 12-week follow-up period. All patients will be educated about PD-related constipation during the run-in period. BYZJ granules and simulated granules will be administered twice daily for 4 weeks to the BYZJ group and the placebo group respectively. Assessments will be performed during run-in period, before the start of treatment (baseline, week 0), and at 4, 8, and 16 weeks. The primary outcome will be measured with the Constipation Severity Instrument, and secondary outcomes will be evaluated with the Patient Assessment of Constipation Quality of Life questionnaire, Bristol Stool Form Scale, Movement Disorders–Unified Parkinson Disease Rating Scale, Nonmotor Symptoms Scale, PD Sleep Scale, Parkinson Fatigue Scale-16. Laxative use (dose and frequency) will also be recorded. Intention-to-treat and per-protocol set analyses will be used to compare symptom improvement between the 2 groups. Any adverse events will be recorded.

**Discussion:**

If found effective and safe, BYZJ formula will be one of Chinese herb to treat constipation and even other nonmotor or motor symptoms in PD patients. The results will sustain the broader use of BYZJ formula in PD.

## 1 Introduction

Constipation is a common non-motor symptom (NMS) of Parkinson disease (PD).^[1,2]^ In China, 64.7% of PD patients experience constipation,^[3]^ and 47.8% use laxatives,^[4]^ which reduces their quality of life. Long-term use of laxatives—especially of the irritant type—can lead to dependence and intestinal dysfunction, which aggravates constipation. A variety of medications for the treatment of PD can cause constipation such as levodopa; conversely, constipation affects intestinal absorption of levodopa, thereby reducing its efficacy and aggravating PD symptoms.^[5]^

Chinese herbal medicines have long been used to treat PD. In recent years, traditional Chinese medicine (TCM) has focused more attention on the treatment of PD-related constipation, with many reports demonstrating that it can be alleviated by certain preparations.^[6,7]^ However, there is a dearth of high-quality clinical studies on this subject.^[8]^

The Chinese herbal formulation Bushen Yisui and Ziyin Jiangzhuo (BYZJ) was developed based on practical experiences from TCM, It is used to regulate bowel movements and alleviate insomnia, fatigue, and anxiety, among other conditions. According to the dialectic principles underlying TCM, constipation in PD is caused by a deficiency in kidney and marrow essence (ie, Qi and Yin).^[7–9]^ Thus, strengthening Qi and Yin and nourishing the kidney and marrow is a potential treatment strategy.

The objective of the present study is to assess the efficacy and safety of BYZJ in the treatment of constipation and other NMS as well as motor symptoms (MS) in PD patients.

## 2 Methods/design

This protocol is reported in accordance with the Standard Protocol Items: Recommendations for Interventional Trials guidelines.^[10]^

## 3 Hypothesis

BYZJ is effective and safe for the treatment of constipation, along with other NMS and MS, in patients with PD.

## 4 Study design

**Figure 1:**
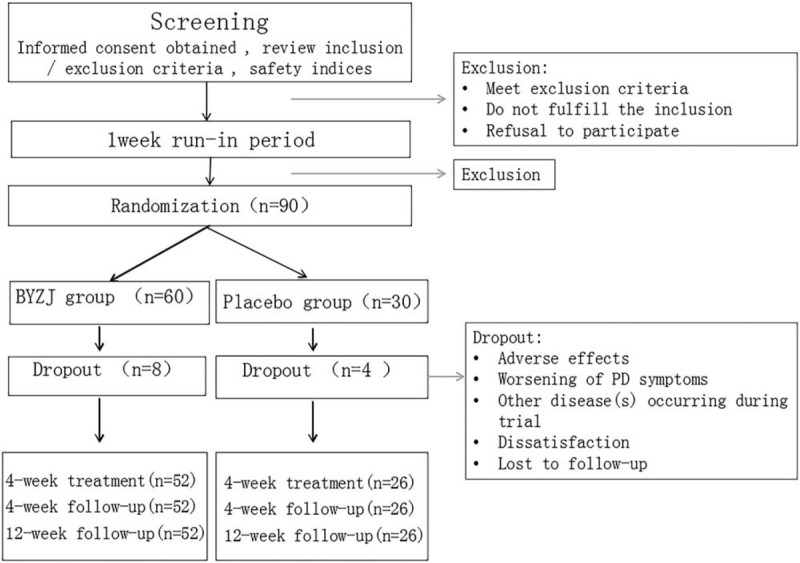
Study design flow chart. The 17-wk study will include a 1-wk run-in period, an intervention time of 4 wk, 4-wk, and 12-wk washout follow-up. Efficacy will be assessed at baseline (week 0) and at 4, 8,16 wk and safety will be assessed at baseline (week 0) and at 4, 16 wk.

This will be a randomized, double-blind, and placebo-controlled multicenter trial. We aim to recruit 90 patients who will be assigned by stratified randomization (in a 2:1 ratio) to intervention (BYZJ) and control (placebo) groups. The total study duration will be 9 weeks, with a 1-week run-in period, an intervention time of 4 weeks, and 4-week and 12-week washout follow-up periods. Efficacy and safety will be assessed at baseline (week 0) and at 4, 8, and 16 weeks (Fig. 1).

## 5 Recruitment, study setting, and participants

Participants will be recruited from the PD outpatient clinic of Beijing Rehabilitation Hospital, Capital Medical University, Xiyuan Hospital of China Academy of Chinese Medical Sciences, Dongzhimen Hospital of Beijing University of Chinese Medicine, and Beijing Longfu Hospital. We will also aim to recruit patients through announcements in the media and advertisements that will be published on official information platforms and the bulletin boards of each study site. Interested prospective subjects will be able to contact investigators using the telephone numbers provided. An investigator will conduct a face-to-face interview with each respondent to explain the study. Those who volunteer to participate will be required to sign appropriate consent forms.

## 6 Inclusion and exclusion criteria

To be included in the trial, participants must meet the following criteria: diagnosed with idiopathic PD according to the 2015 Movement Disorder Society criteria^[11]^; diagnosed with constipation according to the Rome III criteria^[12]^; between 30 and 80 years-of-age; have symptoms that are classified as scale 1 to 4 according to the Hoehn and Yahr scale; provide informed consent and confirm that they will be able to complete the whole treatment; and refrain from participating in other clinical trials during the study and follow-up period. The exclusion criteria are as follows: patients who do not take their medication under the guidance of a doctor or cannot take their medication regularly; patients with Parkinsonism-Plus syndrome or Secondary Parkinson syndrome caused by drugs, metabolic diseases, encephalitis, stroke, brain tumours, carbon monoxide poisoning, and other neurologic degenerative diseases; patients with organic intestinal lesions such as intestinal polyps, intestinal tuberculosis, Crohn disease, tumours, and ulcers; patients with secondary constipation caused by uremia, muscular dystrophy, multiple sclerosis, hypothyroidism and other system diseases; patients with severe heart, brain, liver, kidney, and blood diseases, as well as mental disorders or dementia; patients with serum creatinine, alanine aminotransferase, or aspartate transaminase levels more than twice the normal value; according to the investigators’ judgment, patients who are unable to cooperate throughout the study because of drug abuse or poor adherence (as judged by the investigators); patients who participated in other studies in the previous 2 weeks or are currently participating in other clinical trials, or have taken other TCM preparations in the previous 3 months; pregnant and lactating women; and patients or family members who are unwilling to cooperate or do not agree to sign the informed consent form.

## 7 Sample size

The minimum sample size for statistical significance with a 2-sample 1-sided *t* test was estimated based on the results of a previous study involving the treatment of patients with PD-related constipation.^[13]^ Based on a 2:1 ratio of patients in the BYZJ and placebo groups, the minimum sample size was calculated with formula shown in Eq. (1).: 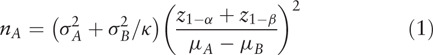


In Equation 1, n represents the sample size of each group, κ = nA/nB; μ represents the overall mean, μA represents the mean of group A, μB represents the mean of group B; σ represents the overall standard deviation, σA represents the standard deviation of group A, σB represents the standard deviation of group B; α represents type 1 errors; and β represents type 2 errors. Application of Eq. (1) showed that 52 are required for the BYZJ group and 26 cases are required for the placebo group. Considering a 15% dropout rate, we plan to recruit 60 and 30 cases for the BYZJ and placebo groups, respectively.

## 8 Randomization and blinding

We plan to use the complete randomization function of SAS 9.13 statistical software (SAS Institute, Cary, NC) to create a table of random numbers that will be printed out and placed in a light-proof envelope and managed by a third party (not the investigators). Under a double-blind design, BYZJ granules and BYZJ simulated granules will be provided to patients in the BYZJ and placebo groups, respectively. Both formulations will be provided by the Dongzhimen Hospital pharmacy and labeled as constipation No. 1 and 2, respectively. In order to ensure blinding, both formulations will have identical packaging. The third party will be responsible for the implementation of blinding and its removal after the trial.

In the event of a potential Suspected Unexpected Serious Adverse Reaction to the trial medication, unblinding will be undertaken by the sponsor in accordance with the regulatory requirements. Unblinding may also be performed at the request of a senior clinician responsible for the care of a trial participant but such requests are likely to occur only in the case of a serious adverse clinical event and are expected to be rare.

## 9 Intervention

### 9.1.1 Run-in period

Subjects will receive health education with regards to constipation by specialist personnel during the 1-week run-in period. We also plan to develop a constipation instruction manual that includes dietary guidance, precautions, and the management of bowel habits.

### 9.1.2 Treatment period

BYZJ granules (Table 1) consist of *Cistanche*, Chinese wolfberry, *Rehmannia glutinosa*, dogwood, raw *Rehmannia*, *Radix Ginseng*, hemp kernel, raw *Astragalus*, *Gastrodia elata*, and raw licorice (Table 1). Patients will be given BYZJ granules (69.5 g twice daily) dissolved in warm water for 30 to 60 min after both breakfast and dinner (approximately 100–150 mL each time) for 4 weeks.

**Table 1:**
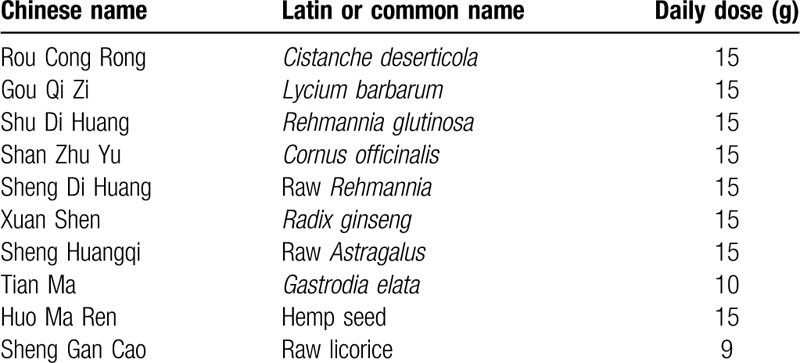
Components of the BYZJ formulation.

The placebo will consist of 95% dextrin and 5% BYZJ granules, and will have the same characteristics as and taste as the BYZJ granules. The placebo will be administered at the same dosage and following the same regimen.

If subjects do not defecate for 3 or more days, we will allow them to take a laxative (eg, Kaiseruna) throughout the trial. The amount of laxatives, and the frequency of their use, will be recorded for each patient.

## 10 Conventional anti-Parkinsonian drugs

The conventional medical treatment plan used by the subjects will need to meet the requirements of the European Federation of the Neurological Societies guideline for the Treatment of PD.^[14]^ Typical drug types include levodopa, dopamine agonists, monoamine oxidase type B inhibitors, catechol-O-methyl transferase inhibitors, amantadine, and anticholinergics.

## 11 Combined medication

Any medication used for comorbidities will be recorded in detail, including the reason for their use, the name of the drug, the dose per administration, the total doses per day, the start date, the date of discontinuation, and the patient's status at the last visit.

## 12 Outcome measures and safety

**Figure 2:**
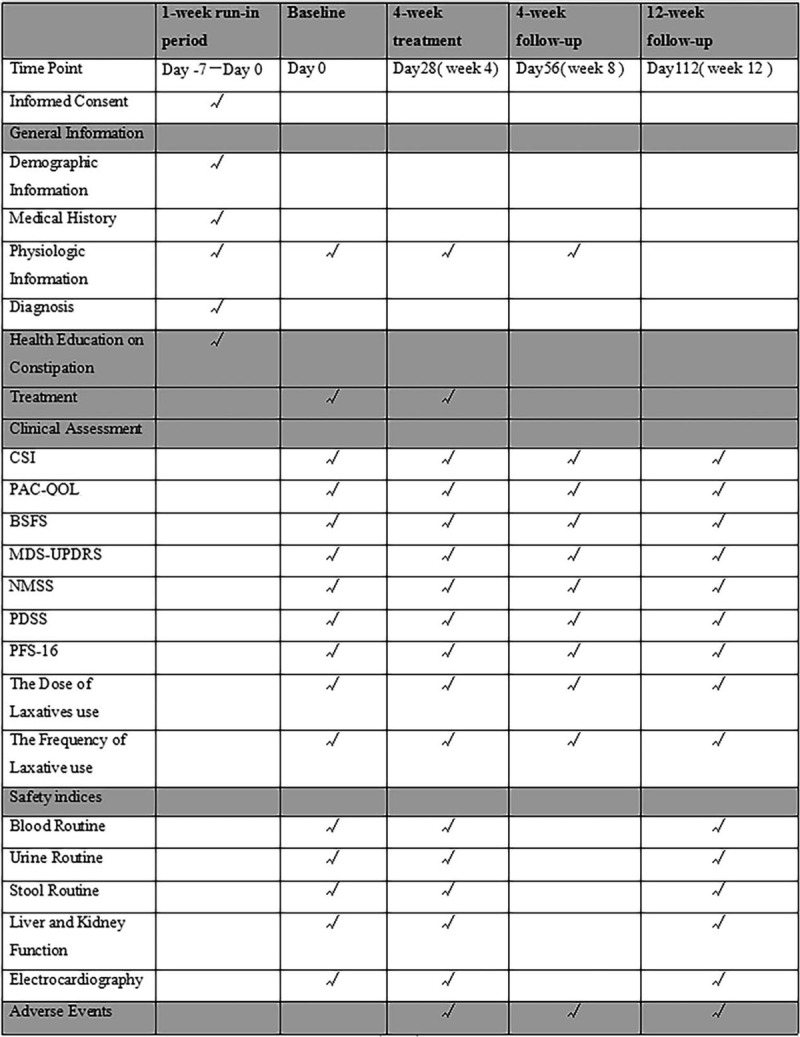
Time schedule of enrollment, interventions, and assessments: In the run-in period, Subjects will receive health education on constipation by specialist personnel and inform consent, General patient information will be collected; At baseline (week 0) and at 4, 8, 16 wk, CSI will be used to assess the severity of constipation; the PAC-QOL questionnaire to evaluate the impact of constipation on the quality of life of patients with PD; and BSFS to assess stool characteristics. The MDS-UPDRS scale will be used to comprehensively evaluate patients’ daily life experience including MS, NMS, and motor complications. The NMSS allows quantitative evaluation of NMS of PD; the PDSS is used to assess sleep quality in PD patients; and PFS-16 measures the severity of fatigue; the Physiologic information and Safety indices will also be tested at baseline (week 0) and at 4, 16 wk; adverse events will be recorded during the research. BSFS = bristol stool form scale, CSI = constipation severity instrument, MDS-UPDRS = the new world movement disorders comprehensive Parkinson disease rating scale, NMS = nonmotor symptoms, NMSS = nonmotor symptoms scale, PAC-QOL = patient assessment of constipation quality of life questionnaire, PDSS = Parkinson disease sleep scale, PFS-16 = Parkinson fatigue scale.

We will collect a range of general information for each patient during the run-in period. The primary and secondary outcomes will be assessed at week 0 (baseline), and then again at weeks 4, 8, and 16. Safety and laboratory indicators will be assessed at week 0 (baseline), and then again at weeks 4 and 16 (Fig. 2). The primary outcome will be the change in constipation severity instrument score. Secondary outcomes include scores for the Patient Assessment of Constipation Quality of Life questionnaire, the Bristol stool form scale, the movement disorders-unified Parkinson disease rating scale, the nonmotor symptoms scale, the Parkinson disease sleep scale, and the Parkinson fatigue scale. We will also record the dose of laxatives and their frequency of use.

We will collect a range of demographic information, including name, sex, age, date of birth, height, weight, education, history of tobacco and alcohol use, family history of PD, occupation, home address, and contact telephone number. We will also record each patient's medical history, including current medical status and past medical history. Physiological data will include pulse, respiration, heart rate, standing blood pressure, and nervous system function. Safety indicators will include routine blood, urine, and stool parameters; liver and kidney function; and electrocardiography parameters. Any adverse events after the start of treatment will be recorded along with incident handling.

## 13 Quality control

Before the start of the study, the investigators will receive standardized training relating to the data collection strategy and guidelines for the different scales that will be used to assess outcomes. During the trial, data will be collected by trained researchers to prevent bias. If the subjects experience the “on-off” phenomenon (In Parkinson patients, the alternating periods of good control (on) and poor control (off) of their symptoms. The on-off phenomenon is often experienced in patients undergoing L-dopa therapy), the visit should be conducted during the “on” period.

## 14 Data management

An electronic data capture (EDC) system, that relies on the clinical research data platform of the Parkinson Medical Center at the Beijing Rehabilitation Hospital, will be used for all data entry and management. Data entry will be carried out by 1 data collector assigned to each center. Participant information will be stored in the EDC system; investigators will access this system using a password. The investigators will also check the case report forms to confirm that they are complete and also check for consistency between the code and the subject's screening entry list. Data will be validated after entry into the EDC system, and the original case will be reviewed again based on these results. Final data will be exported as an Excel (Microsoft, Redmond, WA) spreadsheet.

## 15 Data monitoring

We have already established an independent Data and Safety Monitoring Board (DSMB) for this study that is independent of the sponsor, consisting of a TCM specialist, a Neurologist and a statistician. The DSMB will oversee whether the trial follows the study design and standard guidelines. The neurologist will assess, manage, and classify all adverse events. All serious adverse events will need to be reported to the main researchers, the Ethics Committees, and the DSMB, within 24 hours of occurrence. The DSMB and the main researchers will discuss such issues. The Ethics Committees and the DSMB will have the right to terminate the trial if they deem that such action is appropriate. The role of the statistician is to review the safety data.

## 16 Statistical analysis

SPSS v20.0 software (SPSS Inc., Chicago, IL) will be used for all data processing and analysis. Measurement data will be expressed as mean ± standard deviation. The normality and homogeneity of variance will be tested. Data with a normal distribution and uniform variance will be evaluated with the independent samples *t*-test for between-group comparisons. Data that do not meet these criteria will be evaluated with a nonparametric test. Repeated measures analysis of variance will be used for within-group comparisons (1-way analysis of variance will be used if the data meet the Huynh–Feldt condition; otherwise, a mixed model will be used).

Numerical (count) data will be expressed as percentages. Between-group comparisons will be performed with the *χ*^2^ test. Intent-to-treat and compliance analyses will be performed to compare efficacy between the 2 groups. The last observation carry-forward method will be used for data processing. The significance level for each test will be set at *P* < .05.

An intention-to-treat analysis will be adopted to deal with missing data, including all participants in the analysis based on the initial group allocation. A linear mixed model approach (a direct likelihood estimation method) will be applied to analyze all continuous variables as value is missing at random.

## 17 Ethics approval and consent to participate

The protocol adheres to the guidelines outlined by the Declaration of Helsinki Declaration and the Measures for Ethical Review of Biomedical Research involving Humans,^[15]^ and has been approved by the Ethics Committee of Beijing Rehabilitation Hospital, Capital Medical University. The trial will be conducted under the supervision of the Clinical Trials Center at the Rehabilitation Hospital, Capital Medical University. All participants must provide written, informed consent. All data will be anonymized. Participation is voluntary and patients may leave the study at any time. We will set up multiple communication channels to maintain close contact with participants. Those who are assigned to the placebo group will receive a 4-week supply of BYZJ granules as compensation after the trial has ended.

## 18 Protocol amendments and dissemination

The personal information of all participants will remain private; only relevant codes will be used during statistical analysis. Participant information will be stored for 5 years in the EDC system at the Rehabilitation Hospital Affiliated with Capital Medical University, China. The final data set will be available to the principal investigator and the independent statistician. All participants will be asked if they would like to receive a copy of the outcomes of the study after the invention and an email address or telephone number will be collected to facilitate the distribution of any relevant publications. The participants will be informed of the result of this study after the intervention. The results of this study will be published in a peer-reviewed journal and presented at national and international conferences. We will follow all authorship eligibility guidelines and we do not intend to use professional writers.

## 19 Discussion

### 19.1 Effects of constipation

Constipation is characterized by irregular bowel movements caused by dry feces or excretory difficulty even when feces are not dry. Clinically, patients are classified as having mild, moderate, and severe functional constipation according to the degree of constipation and the impact on quality of life.^[16]^ Constipation is among the most common NMS of PD, with an incidence of 47% to 60%.^[17]^ Moreover, constipation may manifest years before a clinical diagnosis of PD^[18,19]^ and may precede the appearance of typical MS.^[20–24]^ The nonmotor symptoms scale and the autonomic NMS scores of PD patients with constipation are significantly higher than those of patients without constipation.^[25,26]^ Furthermore, research has suggested that constipation is closely related to other NMS such as urinary urgency, nocturia, orthostatic hypotension, and attention deficit.

### 19.2 The pathogenesis of constipation in PD

The pathogenesis of constipation in PD is thought to be related to several factors. Degenerative changes in the enteric nervous system such as the loss of intestinal interstitial dopaminergic neurons in patients with PD are known to affect the control of gastrointestinal motility, thereby weakening intestinal function and leading to constipation.^[27]^ Defecation involves coordination between abdominal, internal anal sphincter, external anal sphincter, and puborectalis muscles; any dysregulation of this activity will reduce gastrointestinal motility, thereby obstructing defecation. Approximately 80% of patients with PD have delayed colonic transit, with a mean transit time of 43 hours to 168 hours compared to 12 hours in normal individuals.^[28]^ PD-related constipation is also a side effect of, or can be aggravated by, anti-Parkinson drugs.^[29]^ For example, levadopa slows gastrointestinal motility; the use of dopaminergic drugs has previously been shown to increase the frequency of constipation.^[30]^ A range of other factors are known to contribute to PD, including overall movement, reduced physical activity, eating habits, reduced water intake, mood, and intestinal dysbiosis.^[31]^

### 19.3 The treatment of PD-related constipation

Western medical treatments for constipation include general-, drug-, and surgical-based treatments, and biofeedback therapy.

#### 19.3.1 General treatment

General treatment options include the replacement or discontinuation of drugs that induce constipation. Patients should also be encouraged to exercise, adopt reasonable eating habits, and increase their fluid and fibre intake. Collectively, these actions can result in regular bowel movements and relieve stress.

#### 19.3.2 Drug treatment

A range of drugs are commonly used to treat PD-related constipation, including laxatives, enemas, suppositories, drugs that promote gastrointestinal motility, and microecological preparations. The long-term intake of irritant laxatives, such as bisacodyl, can lead to colon hypofunction and is not recommended for routine use. Enemas (eg, Kaiser) are administered through the anus but should be used cautiously to avoid dependence. Cisapride is no longer prescribed as this drug can increase the risk of cardiovascular disease. Lubiprostone is known to enhance intestinal secretion although this drug is associated with adverse effects such as nausea, diarrhea, and headache; moreover, the high cost of this treatment discourages adherence in many patients. Microbiological preparations, such as Bifidobacterium viable and triple-viable capsules, and Licheniformis viable capsules, promote bowel movement by reversing intestinal dysbiosis, although they have limited efficacy.

#### 19.3.3 Biofeedback therapy

Biofeedback therapy is a treatment option for constipation caused by pelvic floor muscle dysfunction. This method aims to stimulate defecation through sounds and images; feedback to the brain triggers defecation by restoring the resting potential of the sphincter muscles. The efficacy of this treatment depends on the patient's compliance and the experience of the therapist.^[32]^

#### 19.3.4 Surgical treatment

In rare cases, surgical intervention can represent an option for treating PD-related constipation, although there are strict indications and contraindications for its use.^[33]^ Moreover, surgical treatment has questionable efficacy; recurrence and complications often lead to poor outcomes.

### 19.4 Caveats associated with modern medical treatments for constipation

Although laxative medicines can immediately relieve constipation, their effects may be short-lived, and their long-term use can lead to dependence and colonic melanosis. Furthermore, enema and pelvic floor biofeedback therapy can cause pain and are less effective than drugs. Surgical treatment for constipation remains in its infancy; in addition to its high cost, surgery may not provide long-term relief and patients may experience considerable pain and adverse events.

### 19.5 TCM for constipation in PD

TCM has certain advantages over Western medicine for the treatment of constipation in PD patients because it is based on dialectic therapy and the notion that constipation and PD have the same pathogenic origin. In TCM, kidney essence is fundamental to the body as it strengthens Qi, Yin, and cerebral function to promote agility, strengthen the spirit and bones, and allow smooth stool passage.^[34,35]^ Sluggish movement, involuntary tremor, rigidity, constipation, insomnia, and fatigue in PD are considered as manifestations of a lack of kidney essence (and consequently, a lack of Qi and Yin, as well as cerebral dysfunction). Thus, the management of PD-related constipation should be based on the principle of nourishing the kidney essence by strengthening Qi and Yin.

Enhancing kidney and marrow function by strengthening Qi and Yin can alleviate NMS of PD such as constipation.^[36,37]^ Chinese herbal formulations including Buzhong Yiqi decoction^[38,39]^; Bushen Ziyin Runchang pills^[40]^; and Zuogui pills and Zengye Chengqi decoction^[41]^; can nourish the kidney essence^[40,42]^ while Qi and Yin^[38,39,43]^ can reduce turbidity.^[44]^ Collectively, these formulations have been shown to be effective in treating constipation in patients with PD. Stimulating ganglion cells of the intestinal nerve through acupuncture can also alleviate constipation by regulating intestinal function and enhancing peristalsis in the large intestine.^[45–49]^

Western medicines mostly target MS of PD, but do not improve NMS; they may even exacerbate the problem. These medicines are also associated with many adverse effects and poor long-term outcomes. In contrast, TCM has a good curative effect with regards to PD and address both the MS and NMS of PD when combined.

### 19.6 BYZJ formulation

The BYZJ formulation is derived from the Liuwei Dihuang Wan and Zengye decoction and has been pre-tested for the treatment of constipation, insomnia, and fatigue in patients with PD. Unlike other TCM treatments, BYZJ targets deficiencies in the kidneys, marrow, Qi, and Yin, through kidney tonification, Qi and Yin nourishiment, and turbidity reduction. The BYZJ formulation includes *Cistanche*, medlar, cooked *Rehmannia*, Dogwood, raw *R glutinosa*, black ginseng, hemp kernel, raw Astragalus, *Gastrodia*, and raw licorice. Our proposed randomized, double-blind, and placebo-controlled trial is expected to provide reliable evidence for the efficacy and safety of the BYZJ formulation for the treatment of constipation and other NMS symptoms in PD patients.

## Acknowledgments

The authors thank all participants and researchers at each center for their contributions at each stage of this research.

## Author contributions

**Conceptualization:** Zhaohui Jin, Boyan Fang.

**Funding acquisition:** Zhaohui Jin.

**Methodology:** Boyan Fang.

**Project administration:** Boyan Fang.

**Supervision:** Boyan Fang.

**Writing – original draft:** Zhaohui Jin.

**Writing – review & editing:** Boyan Fang.
